# Methods used in the spatial analysis of tuberculosis epidemiology: a systematic review

**DOI:** 10.1186/s12916-018-1178-4

**Published:** 2018-10-18

**Authors:** Debebe Shaweno, Malancha Karmakar, Kefyalew Addis Alene, Romain Ragonnet, Archie CA Clements, James M. Trauer, Justin T. Denholm, Emma S. McBryde

**Affiliations:** 1 Department of Medicine, University of Melbourne, Melbourne, Victoria Australia; 2 Victorian Tuberculosis Program at the Peter Doherty Institute for Infection and Immunity, Melbourne, Victoria Australia; 3 Department of Microbiology and Immunology, University of Melbourne, Melbourne, Victoria Australia; 4 Research School of Population Health, College of Health and Medicine, The Australian National University, Canberra, Australia; 5 Institute of Public Health, College of Medicine and Health Sciences, University of Gondar, Gondar, Ethiopia; 6 Burnet Institute, Melbourne, Australia; 7 Curtin University, Bentley, Western Australia Australia; 8 School of Public Health and Preventive Medicine, Monash University, Melbourne, Australia; 9 Australian Institute of Tropical Health and Medicine, James Cook University, Townsville, Queensland Australia

**Keywords:** Spatial analysis, Tuberculosis, Genotypic cluster

## Abstract

**Background:**

Tuberculosis (TB) transmission often occurs within a household or community, leading to heterogeneous spatial patterns. However, apparent spatial clustering of TB could reflect ongoing transmission or co-location of risk factors and can vary considerably depending on the type of data available, the analysis methods employed and the dynamics of the underlying population. Thus, we aimed to review methodological approaches used in the spatial analysis of TB burden.

**Methods:**

We conducted a systematic literature search of spatial studies of TB published in English using Medline, Embase, PsycInfo, Scopus and Web of Science databases with no date restriction from inception to 15 February 2017.

The protocol for this systematic review was prospectively registered with PROSPERO (CRD42016036655).

**Results:**

We identified 168 eligible studies with spatial methods used to describe the spatial distribution (*n* = 154), spatial clusters (*n* = 73), predictors of spatial patterns (*n* = 64), the role of congregate settings (*n* = 3) and the household (*n* = 2) on TB transmission. Molecular techniques combined with geospatial methods were used by 25 studies to compare the role of transmission to reactivation as a driver of TB spatial distribution, finding that geospatial hotspots are not necessarily areas of recent transmission. Almost all studies used notification data for spatial analysis (161 of 168), although none accounted for undetected cases. The most common data visualisation technique was notification rate mapping, and the use of smoothing techniques was uncommon. Spatial clusters were identified using a range of methods, with the most commonly employed being Kulldorff’s spatial scan statistic followed by local Moran’s *I* and Getis and Ord’s local Gi(d) tests. In the 11 papers that compared two such methods using a single dataset, the clustering patterns identified were often inconsistent. Classical regression models that did not account for spatial dependence were commonly used to predict spatial TB risk. In all included studies, TB showed a heterogeneous spatial pattern at each geographic resolution level examined.

**Conclusions:**

A range of spatial analysis methodologies has been employed in divergent contexts, with all studies demonstrating significant heterogeneity in spatial TB distribution. Future studies are needed to define the optimal method for each context and should account for unreported cases when using notification data where possible. Future studies combining genotypic and geospatial techniques with epidemiologically linked cases have the potential to provide further insights and improve TB control.

**Electronic supplementary material:**

The online version of this article (10.1186/s12916-018-1178-4) contains supplementary material, which is available to authorized users.

## Background

*Mycobacterium tuberculosis* (*Mtb*) transmission often occurs within a household or small community because prolonged duration of contact is typically required for infection to occur, creating the potential for localised clusters to develop [1]. However, geospatial TB clusters are not always due to ongoing person-to-person transmission but may also result from reactivation of latent infection in a group of people with shared risk factors [1, 2]. Spatial analysis and identification of areas with high TB rates (clusters), followed by characterisation of the drivers of the dynamics in these clusters, have been promoted for targeted TB control and intensified use of existing TB control tools [3, 4].

TB differs from other infectious diseases in several ways that are likely to influence apparent spatial clustering. For example, its long latency and prolonged infectious period allow for significant population mobility between serial cases [5]. Thus, *Mtb* infection acquired in a given location may progress to TB disease in an entirely different region, such that clustering of cases may not necessarily indicate intense transmission but could rather reflect aggregation of population groups at higher risk of disease, such as migrants [6]. Similarly, *Mtb* infection acquired from workplaces and other congregate settings can be wrongly attributed to residential exposure, as only an individual’s residence information is typically recorded on TB surveillance documents in many settings [7, 8].

Identifying heterogeneity in the spatial distribution of TB cases and characterising its drivers can help to inform targeted public health responses, making it an attractive approach [9]. However, there are practical challenges in appropriate interpretation of spatial clusters of TB. Of particular importance is that the observed spatial pattern of TB may be affected by factors other than genuine TB transmission or reactivation, including the type and resolution of data and the spatial analysis methods used [10]. For instance, use of incidence data versus notification data could give considerably different spatial pattern [11], as the latter misses a large number of TB cases and could be skewed towards areas with better access to health care in high-burden settings [12, 13]. Thus, spatial analysis using notification data alone in such settings could result in misleading conclusions.

Similarly, the type of model used and the spatial unit of data analysis are important determinants of the patterns identified and their associations [14–16]. That is, different spatial resolutions could lead to markedly different results for the same dataset regardless of the true extent of spatial correlation [15, 17, 18] and the effect observed at a regional level may not hold at the individual level (an effect known as the ecological fallacy) [19]. Therefore, we aimed to review methodological approaches used in the spatial analysis of TB burden. We also considered how common issues in data interpretation were managed, including sparse data, false-positive identification of clustering and undetected cases.

## Methods

### Data source and search strategy

Our search strategy aimed to identify peer-reviewed studies of the distribution and determinants of TB that employed spatial analysis methods. In this review, studies were considered spatial if they incorporated any spatial approaches (e.g. geocoding, spatial analysis units, cluster detection methods, spatial risk modelling) into the design and analysis of the distribution, determinants and outcomes of TB [20]. We searched Medline, Embase, Web of Science, Scopus and PsycInfo databases from their inception to 15 February 2017 using a combination of keywords and medical subject headings (MeSH) pertaining to our two central concepts: tuberculosis and space. We refined search terms related to the latter concept after reviewing key studies, including a previous systematic review not limited to TB [21]. The full search strategy was adapted to the syntax of the individual database from the following conceptual structure: (tuberculosis OR multidrug-resistant tuberculosis) AND (spatial analysis OR geographic mapping OR spatial regression OR spatiotemporal analysis OR spatial autocorrelation analysis OR geography OR geographic distribution OR geographic information system OR geographically weighted regression OR space-time clustering OR ‘spati*’ OR ‘hotspots’ OR cluster analysis) and is provided in the Appendix. Studies targeted to special populations (e.g. homeless, migrants, HIV-infected persons) and that considered the entire population of a region were permitted. Additional papers were also identified through hand searching the bibliographies of retrieved articles and from suggestions from experts in the field.

### Eligibility, and inclusion and exclusion criteria

We included peer-reviewed papers that incorporated the spatial analysis approaches described above in the study of TB. After exclusion of duplicates, titles and abstracts were screened by two researchers (DS and MK) to identify potentially eligible studies. Of these papers, articles were excluded hierarchically on the basis of article type, whether the method used could be considered spatial or not and the outcomes assessed. No exclusions were made on the basis of the outcome reported, with studies that considered incidence, prevalence or any TB-related health outcome included. Studies were excluded if the language of the publication was not English, the report was a letter, conference abstract or a review or only reported the temporal (trend) of TB. Spatial studies of non-tuberculous mycobacteria, non-human diseases and population immunological profiles were also excluded. Full-text articles were excluded if they did not provide sufficient information on the spatial analysis techniques employed. There were no exclusions based on study setting or anatomical site of disease.

### Data extraction and synthesis

Three independent reviewers (DS, MK, KAA) performed data extraction using pretested data extraction forms and stored these in a Microsoft Excel 2016 spreadsheet (Microsoft Corporation, Redmond, Washington, USA). Disagreements were resolved by consensus. The following information was extracted from each paper: country, publication year, study aim, data type (notifications or survey), type of TB disease (smear-positive pulmonary, smear-negative pulmonary and extrapulmonary), geographic level, spatial methods (map types, cluster detection methods, statistical regression methods, spatial lag, spatial error, spatial smoothing techniques), time scale and outcomes reported (whether quantification of TB cases or TB-related health outcomes, such as mortality, default from care, disability-adjusted life years (DALYs) and key conclusions). In studies which combined geospatial methods with genotypic clustering methods, we also extracted the genotypic cluster identification methods. Spatial analysis techniques were categorised as either visualisation (mapping), exploration (using statistical tests to identify spatial clusters) or statistical modelling [19, 22]. Counts and proportions were primarily used to summarise study findings. The protocol for this systematic review was prospectively registered with PROSPERO (CRD42016036655). Although we adhered to our original published protocol, here we additionally describe the importance of genotypic methods and the application of spatial methods in informing public health interventions in response to requests during peer review.

## Results

### Study characteristics

**Fig. 1:**
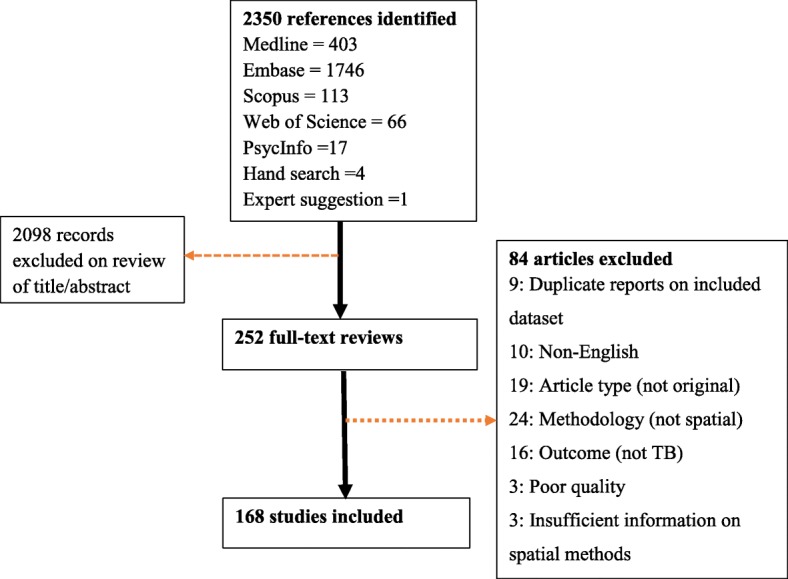
Study inclusion flow chart

A total of 2350 records were identified from the electronic searches, of which 252 full-text articles were assessed. Of these, 168 articles met all inclusion criteria and were included in the final narrative synthesis (Fig. 1). Using a cutoff of 100 TB cases per 100,000 population in reported incidence in 2016, 111 (66%) of the studies were from low-incidence settings.

All references returned by the search strategy were from the period 1982 to 2017, with 71% published from 2010 onwards (Additional file 1: Figure S1). Earlier studies (predominantly in the 1980s and 1990s) tended to be descriptive visualisations, while studies in the last two decades frequently incorporated cluster detection and risk prediction. More recently, a range of statistical techniques including Bayesian statistical approaches and geographically weighted regression have become increasingly popular.

### Key objectives of included studies

Spatial analysis was applied to address a range of objectives (Table 1), with the commonest ones including description of the distribution (*n* = 135), statistical analysis of spatial clustering (*n* = 73) and analysis of risk factors and risk prediction (*n* = 64). Spatial methods were also used to determine the relative importance of transmission by comparison to reactivation as a driver of TB incidence (*n* = 25), the effect of TB interventions (*n* = 2), barriers to TB service uptake (*n* = 2), spatial distribution of TB-related health outcomes (mortality, default, hospitalisation) (*n* = 5), spatial pattern of TB incidence among people living with HIV (PLHIV) (*n* = 4), HIV-related TB mortality (*n* = 4), multidrug-resistant TB (MDR-TB) drivers (*n* = 1), TB outbreak detection (*n* = 3) and drivers of spatial clustering (including the role of congregate settings, such as social drinking venues and schools) (*n* = 30).

**Table 1:** Application areas of spatial methods in TB studies

Spatial method application areas	Methods used	References
Spatial TB distribution or spatial clustering	Dot maps, rate maps, thematic maps, Moran’s *I*, GetisOrd statistic, NNI Besag and Newel statistic, *k*-functions, spatial scan statistic	[1, 2, 7, 8, 12, 16, 23–41, 44–49, 51–54, 57–72, 75, 93–95, 99, 100, 102–176]
Risk factors	Bayesian CAR models, regression models (with or without including spatial terms), GWR, PCA, mixture models, spatial lag models	[8, 12, 33, 36, 38, 40, 42–44, 46–52, 58, 59, 62, 70, 71, 93, 94, 99–102, 104, 111, 112, 116, 117, 120, 123, 125, 127–129, 131, 136, 137, 141–143, 145, 148, 149, 156, 161, 164, 176–189]
Monitoring spatiotemporal TB trends	Temporal trend maps	[27, 36–39]
Intervention evaluation	Distance map, kernel density map	[73, 74]
Barriers to TB care	Rate map, dot map, travel time map, distance map	[12, 187]
TB program performance	Map (time to detection)	[184]
HIV-related TB incidence	Rate map, dot map, spatial scan statistic	[40, 166, 186, 190]
TB treatment outcomes	Spatial empirical Bayes smoothing, kernel density maps, spatial scan statistic, spatial regression	[152, 155, 179, 183, 191]
Mortality related to TB/HIV coinfection	Rate map, thematic maps, Moran’s *I* and spatial regression	[42, 43, 174, 192]
Transmission	Dot maps (congregate settings)	[54, 55, 193]
	Dot maps (cases)	[7, 8]
	Geospatial and genotypic clustering methods	[1, 2, 25, 28, 47, 57, 59–72, 93–95, 169, 194]
Methodological	Spatial scan statistic	[25]
TB outbreak detection	Spatial scan statistic	[1, 25, 28]
Prevalence estimation	Model-based geostatistics	[80]
Drivers of MDR-TB	*k*-function	[35]

*NNI* nearest neighbourhood index, *CAR models* conditional autoregressive models, *GWR* geographically weighted regression, *PCA* principal component analysis, *HIV* human immunodeficiency virus, *MDR-TB* multidrug-resistant TB

### Types of TB disease analysed

Spatial analysis was most commonly conducted on data for all types of TB (i.e. without distinction between pulmonary or extrapulmonary; *n* = 121), followed by pulmonary TB only (*n* = 28) and smear-positive pulmonary TB only (*n* = 13). Spatial analysis of multidrug-resistant TB (MDR-TB) and extensively drug-resistant TB (XDR-TB) was reported in 15 studies and one study respectively.

### Data used and scale of analysis

Nearly all studies used retrospective TB program data (notifications), with the exception of five studies that used prevalence surveys and two prospectively collected data. None of the studies using notification data accounted for undetected/unreported cases. In all included studies, spatial analysis of TB was based on the individual’s residence, except for three studies that explored the effect of exposure from social gathering sites.

Spatial analysis was generally done using data aggregated over administrative spatial units (*n* = 131), but the scale of aggregation differed markedly. Common spatial scales included census tract (*n* = 20), district (*n* = 15), postal code (*n* = 15), county (*n* = 15), neighbourhood (*n* = 10), health area (*n* = 7), municipality (*n* = 11), state (*n* = 7), province (*n* = 6), local government area (LGA) (*n* = 4) and ward (*n* = 4). Data were analysed at the individual level in 37 studies, while three studies were reported at a continent and country scale.

### Methods in the spatial analysis of TB

Table 2 shows the range of spatial methods used. Spatial analysis was used to visualise patterns (*n* = 154), explore spatial clusters (*n* = 73) and identify risk factors for clustering (*n* = 64), with risk prediction undertaken by 11 studies. Of the included studies, six did not explicitly report any of these methods but reported statistical results that implied the use of these methods.

**Table 2:** Spatial methods used in spatial analysis of tuberculosis (*n* = 168)

Method category	Method	Number	References
Visualisation	Rate map	63	[12, 16, 23, 26, 27, 29–34, 37, 41, 44–46, 48, 51, 52, 57, 58, 60, 61, 70, 100, 102, 103, 105, 106, 120, 123–146, 164, 165, 170, 173–176, 195, 196]
	Dot map	37	[2, 7, 8, 35, 40, 47, 53, 54, 59, 66, 67, 72, 73, 75, 95, 107–122, 158, 166, 169, 178, 191, 197]
	SMR map	12	[38, 49, 99, 100, 124, 126, 127, 129, 138, 142, 148, 149]
	Kernel density map	7	[35, 37, 62, 93, 120, 147, 171]
	Case counts maps	3	[108, 167, 172]
	Others*	17	[16, 24, 50, 60, 62, 63, 68, 71, 99, 100, 103, 104, 116, 148, 166, 168, 185, 198]
Spatial cluster analysis	Global Moran’s *I*	28	[16, 26, 34, 37, 39, 44, 48, 49, 51, 58, 65, 93, 100, 102, 123, 126, 128, 131, 133, 135, 138, 139, 145, 150, 161, 180, 188, 199]
	Local Moran’s *I*	14	[16, 41, 44, 49, 51, 93, 100, 123, 126, 131, 135, 138, 145, 192]
	Kulldorff’s spatial scan statistic	43	[1, 2, 23–32, 40, 57, 63, 64, 70, 71, 94, 109–111, 119, 120, 130, 135, 138, 139, 141, 151–160, 163, 164, 166, 191]
	GetisOrd statistic	12	[2, 16, 26, 39, 49, 54, 65, 93, 104, 131, 139, 161]
	*k*-NN	8	[35, 53, 69, 72, 93, 114, 122, 163]
	*k*-function	6	[35, 62, 93, 116, 117, 147]
	Besag and Newell statistic	2	[125, 145]
Statistical modelling	Bayesian CAR models	7	[38, 44, 49, 99, 101, 127, 148]
	Geographically weighted regression	6	[16, 50, 93, 102–104]
	Mixture modelling	2	[142, 149]
	Conventional logistic	15	[8, 40, 70, 71, 94, 95, 111, 112, 120, 141, 161, 177, 178, 187, 189]
	Conventional Poisson	5	[46, 125, 136, 145, 156]
	Conventional linear	5	[12, 47, 129, 137, 176]
	Negative binomial	1	[164]
	Factor analysis	6	[50, 103, 117, 143, 146, 170]
	Regression models with spatial terms	9	[42, 48, 51, 58, 100, 116, 128, 131, 188]
	Spatial prediction	11	[38, 42, 43, 62, 80, 99, 101, 127, 131, 148, 181]

*SMR* standardised morbidity ratio, *k-NN k*-nearest neighbourhood test, *CAR* conditional autoregressive

*Includes maps of disability-adjusted life years (DALYs), survival time, factor scores, probability maps, proportion of cases and regression coefficients

### Data visualisation

Data visualisation was the most consistently applied technique, with 154 of the studies using at least one data visualisation method to present TB distribution and/or risk factor patterns across space (Table 1). The TB incidence rate was the commonest indicator mapped (*n* = 63), followed by event maps (*n* = 37), which were smoothed using kernel density in seven studies. Data visualisation was based on standardised morbidity ratios (SMR) in 12 studies. Five studies reported maps of trends in TB incidence over time, and thematic maps were used in nine to consider the impact of risk factors on TB incidence by displaying the spatial distribution of other variables. Variables plotted included climate (*n* = 1), socioeconomic factors (*n* = 5), diabetes (*n* = 1) and obesity (*n* = 1).

#### Approaches used to account for data sparseness

TB is a relatively rare disease at the population level, and burden is typically expressed in terms of cases per 100,000 population. Various approaches were used to account for this sparseness in the number of cases, such as aggregating cases over administrative geographic levels and over time periods (ranging from 1 to 25 years).

An alternative approach was rate smoothing, although this practice was rare, despite the fact that TB rates were the commonest indicators mapped. In the included studies, smoothed rates were used in six (4%) studies. Similarly, of 12 studies that analysed SMRs, smoothed SMRs were presented in seven. In the included studies, several different data smoothing techniques were used, including fully Bayesian (*n* = 8), empirical Bayes (*n* = 4) and spatial empirical Bayes (*n* = 5). A significant number of visualisation reports (*n* = 30) were not complemented by hypothesis testing, either by exploration methods or modelling approaches. In 12 studies (7%), maps were not presented, but a narrative description of TB burden or a tabular presentation of TB distribution by administrative unit was described.

### Spatial cluster (hotspot) identification

Use of at least one spatial cluster identification method was reported in 73 (43%) studies, with Kulldorff’s spatial scan statistic used most frequently (*n* = 43), followed by Local Moran test (*n* = 14) and Getis and Ord’s local Gi(d) statistic (*n* = 12). Nearest neighbour index (NNI), *k*-function and Besag and Newell methods were reported in eight, six and two studies respectively (Table 1). The presence of overall area-wide heterogeneity was assessed most often using global Moran *I* (*n* = 28). In three studies, no globally significant spatial autocorrelation was seen, although there was spatial clustering locally. Although studies used data aggregated over various spatial scales, only one evaluated the impact of spatial scale on the hotspot detection performance of the spatial scan statistic. Use of individual address-level data improved the sensitivity of the spatial scan statistic compared to data aggregated at the administrative level.

Simultaneous use of two spatial cluster detection methods was reported in 11 studies and showed differences in hotspot identification that ranged from complete disagreement to some degree of similarity (Table 3).

**Table 3:** Comparisons of spatial clusters from multiple cluster identification methods

Author, year	Methods	Outcome	Conclusion
Alene, K, 2017 [49]	Local Moran’s *I* Getis and Ord	Clustered Clustered	50% similarity (two non-significant clusters identified by LISA)
Álvarez-Hernández, G., et al. 2010 [145]	Local Moran’s *I* Besag and Newell	No significant Clustered	Widely conflicting
Dangisso M, et al. 2015 [26]	Getis and Ord Spatial scan statistic	Clustered Clustered	Similar overall pattern, but marked differences by years
Feske, M., et al. 2011 [93, 178]	Getis and Ord GWR residuals	Clustered Heterogeneous	Similar overall pattern, but some local differences
Ge E, et al. 2016 [139]	Getis and Ord Spatial scan statistic	Clustered Clustered	Similar overall pattern, but differences in some locations and across time
Haase I, et al. 2007 [2]	Hotspot analysis SaTScan	Clustered Clustered	Similar overall pattern, but some local differences
Hassarangsee S, et al. 2015 [138]	LISA Spatial scan statistic	Clustered Clustered	Very similar, but not identical
Li L, et al. 2016 [135]	LISA Spatial scan statistic	No significant cluster, Clustered	Widely conflicting
Maceiel ELN, et al. 2010 [131]	LISA, Getis and Ord Model prediction	Clustered Heterogeneous	Widely conflicting
Wubuli A, et al. 2015 [16]	LISA Getis and Ord	Clustered Clustered	Similar overall pattern, but some local differences
Wang T, et al 2016 [102]	Spatial scan statistic Getis and Ord	Clustered Clustered	Similar overall pattern, but some local differences

*GWR* geographically weighted regression; *LISA* local indicators of spatial association

#### False-positive clustering

Not all spatial clusters are true clusters. False-positive clusters can arise from various sources, including data and methods used, and unmeasured confounding. Given that notification data were by far the most commonly used data source in the spatial analyses reviewed here, it could not be determined if these clusters represented true clusters of tuberculosis incidence or if they were caused by factors such as pockets of improved case detection. The role of differential TB detection has been documented in some studies from low-income settings, where increased spatial TB burden was linked to improved health care access [12].

In addition, rate was the commonest disease indicator used for disease mapping, as well as cluster detection in this study. As described earlier, rates are liable to stochasticity and can lead to false-positive clustering. However, rate smoothing and stability (sensitivity) analysis of clusters identified using rates was done in only a few studies [23, 24]. This remains an important area of consideration in the future spatial analysis of TB.

### Spatiotemporal analysis

#### Temporal scale

In the spatial analysis of TB, the time window is an important dimension that influences the spatial pattern of TB [25]. As TB is relatively a rare disease at the population level and has a long incubation period, detection of apparent spatial clusters requires a longer time scale than for acute infectious diseases that may form spatial clusters within days of the start of outbreak. Because of this, the included studies were based on cases that accumulated over considerable time periods, ranging from 1 to 25 years, with use of data aggregated over 5 years being the most frequent practice (20%).

#### Approaches

Generally, two approaches were used in the space-time cluster analysis of TB. The first uses classical space-time clustering using algorithms which scan space over a changing time window, such as Kulldorff’s spatial scan statistic [23, 25–29]. The second approach is to account for the temporal dimension by repeating the spatial analysis for each time unit [26, 30–35]. In some studies, spatial patterns in temporal trends of TB incidence were determined as increasing or decreasing [27, 36–39].

### Spatial statistical modelling

Different statistical modelling approaches were used to describe the relationship between TB and ecological factors in 65 (39%) studies, including nine spatially explicit models using Bayesian approaches. Conditional autoregressive (CAR) models were used in nine models to account for spatial correlation. Classical regression models were used in 33, while non-Bayesian spatial regression models were reported in 12.

Of the regression models that evaluated the effect on model fit of including spatial structure (spatial error or spatial lag), the inclusion of spatial structure improved the performance of the model in seven studies and failed to do so in two (based on deviance information criteria). Spatial lag was explicitly modelled in seven studies and highlighted the significant influence of neighbouring locations on TB distribution.

Traditional models including a Bayesian approach assumed a stationary relationship between TB and its spatial covariates and hence imposed a single (global) regression model on the entire study area. Only six studies used a geographically weighted regression (a local regression model) to accommodate variation in the association between TB and its risk factors from place to place and showed spatially varying (non-stationary) effects (*n* = 6). Other models used included mixture modelling (*n* = 2) and factor analysis using principal component analysis (PCA) (*n* = 4).

### Results from spatial analysis

#### Geographic distribution of TB

The geographic distribution of TB was heterogeneous in all included studies both from low- and high-incidence settings, although no formal hypothesis testing was presented in 55 (33%). An exception was one study from South Africa that reported no significant clustering of cases among HIV patients on ART [40]. Spatial analysis was also used to describe the drivers of drug-resistant tuberculosis, with tighter spatial aggregation of MDR-TB cases compared with non-MDR cases taken as evidence of transmission of MDR-TB [41].

Spatial analyses into both HIV and TB investigated outcomes including HIV-associated TB incidence (*n* = 4) and spatial patterns of TB/HIV-related mortality (*n* = 4). All such studies revealed significant spatial heterogeneity. TB/HIV-related mortality in children was linked to areas with low socio-economic status and maternal deaths [42, 43].

Spatial methods used to study the impact of community-based TB treatment showed marked improvement in access compared to health facility-based treatment approaches (*n* = 1), and similar studies demonstrated travel time and distance to be important barriers to TB control (*n* = 2).

#### Correlations with social and environmental factors

The observed spatial patterns of TB were consistently linked to areas with poverty (*n* = 14), overcrowding and non-standard housing (*n* = 9), ethnic minority populations (*n* = 3), population density (*n* = 2), low education status (*n* = 2), health care access (*n* = 3) and immigrant populations (*n* = 5). However, a minority of studies have also found conflicting or non-significant associations between TB and poverty [44–46], population density [47–49] and unemployment [45, 47].

Four studies (including three from China) examined the correlation of climatic factors with TB incidence, with conflicting results. Two province-level studies in China using data from different time periods found TB burden to be associated with increasing annual average temperature [33, 50], although correlation with humidity was conflicting. Positive associations were observed with average precipitation [33, 50] and with air pressure [33] in these studies, while inverse associations were observed with sun exposure [50] and with wind speed [33]. In contrast, a county-level study which used average monthly climate data within a single province of China found the reverse, with temperature, precipitation, wind speed and sunshine exposure showing associations in the opposite direction [51]. A study that compared TB incidence between regions with different climatic conditions showed higher incidence at dry regions and low incidence in humid regions [52].

#### Space-time analysis to detect TB outbreaks

Studies reporting the application of the spatial methods in the early identification of TB outbreak were uncommon. Space-time TB studies using retrospective surveillance data in the USA found that the spatial scan statistic and other methods could effectively detect outbreaks months before local public authorities became aware of the problem [25, 28]. However, as space-time clusters of TB can be due to either ongoing transmission or reactivation, characterising the drivers that resulted in the spatial clustering is essential. Findings from studies which compared the timeliness and accuracy of space-time clusters in identifying TB outbreaks varied with spatial resolution and the background population, with two studies from the USA detecting ongoing outbreaks [25, 28], in contrast to false alarms due to reactivation TB among immigrants in a study from Canada [1].

#### Spatial analysis of the source of TB infection

Spatial methods were also used to determine the role of households and congregate settings (e.g. social gathering venues, schools) on TB transmission risk (Table 1). The role of the household was determined by cross-referencing child and adolescent TB infection or disease with adult TB in two studies [7, 8]. In these studies, the importance of household exposure declined with the age of the child, such that TB disease or infection was related to residential exposure to adult TB in younger children but not adolescents.

Congregate settings, which pose increased transmission risk, were identified using multiple techniques that included linking TB cases to social gathering places [53] and mapping the distribution of rebreathed air volume (RAV) [54] (including grading these settings based on TB transmission principles [55]). These approaches identified schools and social gathering sites as high-risk areas.

#### Identifying local drivers

Recent transmission is a critical mechanism driving local TB epidemiology in high-burden settings, while reactivation of remotely acquired infection is thought to predominate in most low-endemic settings [4, 56]. Geospatial clusters may reflect increased disease risk due to geographic proximity, which may correspond to recent transmission‚ or reactivation of latent TB infection in an aggregate of individuals infected elsewhere or both [57]. In the reviewed studies, spatial methods coupled with other methods were used to identify which of these two mechanisms drives local TB epidemiology in the following three ways.

##### Combining spatial clusters with cohort clustering:

TB clustering can occur from ongoing transmission or from reactivation of latent infection among high-risk subgroups due to shared characteristics such as similar country of birth rather than a shared transmission network, a phenomenon known as cohort clustering. Cohort cluster analysis is used to identify selected high-risk population subgroups for targeted interventions based on the relative TB incidence they bear. The Lorenz curve is a simple visualisation tool that compares the clustering (inequality) in the subgroup of interest across regions and over time. One study, which combined such cohort (birth country) cluster analysis using the Lorenz curve of inequality with spatial cluster analysis [31] revealed colocation of these cluster types, suggesting the presence of both transmission and reactivation. Spatial clusters among foreign-born persons covered too large an area compared to clusters among the locally born to be consistent with direct person-to-person transmission. In addition, spatial modelling was also applied to differentiate the role of transmission from reactivation by assessing spatial dependence. The presence of spatial dependence (autocorrelation) was taken to indicate transmission, while its absence was considered to indicate reactivation [58].

##### Combining spatial and genotype clustering:

Genotypic clustering of TB may be used as a proxy for recent transmission, such that geospatial clusters in which cases are genotypically clustered may be taken as stronger evidence for locations where recent transmission has occurred. These approaches were combined to quantify the role of recent transmission and determine geographical locations of such transmission in 25 studies. This was done either by determining the spatial distribution of genotypic clusters [25, 28, 59–69] or by assessing the genotypic similarity of cases contained within geospatial clusters [2, 57, 65, 70, 71].

The findings from these studies varied considerably by the country and sub-population studied (locally born versus immigrants) (Table 4). Genotypic clusters were spatially clustered in many studies, providing evidence of recent local transmission. In some studies, cases in geospatial clusters were less likely to be dominated by genotypically similar cases (i.e. were dominated by unique strains) than cases outside the geospatial clusters, implying spatial aggregation of reactivation TB [57]. This finding highlights that geospatial hotspots in low TB incidence settings are not necessarily areas of recent transmission and spatial clustering may be primarily mediated by social determinants, such as migration, HIV and drug abuse [57].

**Table 4:** Overlap between spatial and molecular clustering

Authors	Country	Genotyping methods	Findings
Bishai WR, et al. 1998 [95]	USA	IS6110-RFLP and PGRS	Genotypic clusters with epidemiologic links were spatially clustered but 76% of DNA clustered cases lack epidemiologic links.
Mathema B, et al. 2002 [169]	USA	IS6110-RFLP and spoligotyping	Genotypic clusters showed spatial aggregation
Richardson M, et al. 2002 [72]	South Africa	IS6110-RFLP and spoligotyping	Spatial aggregation of genotypic clusters was limited
Nguyen D, et al. 2003 [69]	Canada	IS6110-RFLP and spoligotyping	Genotypically similar cases were not more spatially clustered than genotypically unique cases
Moonan P, et al. 2004 [61]	USA	IS*6110*-RFLP and spoligotyping	Genotypic clusters were spatially heterogeneous
Jacobson L, et al. 2005 [59]	Mexico	IS*6110*-RFLP and spoligotyping	Spatial patterns were similar for both cases categorised as reactivation or recent transmission
Haase I, et al. 2007 [2]	Canada	IS*6110*-RFLP and spoligotyping	In spatial TB clusters of immigrants, there was significant genotype similarity
Higgs B, et al. 2007 [25]	USA	IS*6110*-RFLP and PGRS	Space-time clusters contained genotypic clusters
Feske ML, et al. 2011 [93, 178]	USA	IS*6110*-RFLP and spoligotyping	Genotypically clustered cases were randomly distributed across space
Evans JT, et al. 2011 [66]	UK	Spoligotyping and MIRU-VNTR	Genotypic clusters showed spatial aggregation
Nava-Aguilera E, et al. 2011 [67]	Mexico	Spoligotyping	Genotypic clusters were not spatially aggregated
Prussing C, et al. 2013 [57]	USA	Spoligotyping and 12- MIRU-VNTR	Cases in geospatial clusters were equally or less likely to share similar genotypes than cases outside geospatial clusters
Tuite AR, et al. 2013 [94]	Canada	Spoligotyping and 24-MIRU-VNTR	The proportion of cases in genotypic clusters was five times that seen in spatial clusters (23% vs 5%)
Kammerer JS, et al. 2013 [28]	USA	Spoligotyping and 12-MIRU-VNTR	Genotypically similar cases were spatially clustered
Verma A, et al. 2014 [1]	Canada	IS*6110*-RFLP and Spoligotyping	Space-time clusters contained few or no genotypically similar cases
Izumi K, et al. 2015 [65]	Japan	IS*6110*-RFLP	Both genotypically similar and unique strains formed spatial hotspots
Chamie G, et al. 2015 [194]	Uganda	Spoligotyping	Genotypic clusters shared social gathering sites (clinic, place of worship, market or bar)
Chan-Yeung M, et al. 2005 [47]	Hong Kong	IS*6110*-RFLP	Spatial locations of genotypic clusters and unique cases did not differ by their sociodemographic characteristics
Gurjav U*,* et al. 2016 [70]	Australia	24-MIRU-VNTR	Spatial hotspots were characterised by a high proportion of unique strains; less than 4% of cases in spatial clusters were genotypically similar
Ribeiro FK, et al. 2016 [62]	Brazil	IS*6110*-RFLP and Spoligotyping	Genotypic clusters were spatially clustered
Saavedra-Campos M, et al. 2016 [71]	England	24-MIRU-VNTR	10% of cases clustered spatially and genotypically
Seraphin MN, et al. 2016 [64]	USA	Spoligotyping and 24-MIRU-VNTR	22% of cases among USA-born and 5% among foreign-born clustered spatially and genotypically
Yuen CM, et al. 2016 [68]	USA	Spoligotyping and 24-MIRU-VNTR	Genotype clustered cases were spatially heterogeneous
Yeboah-Manu D, et al. 2016 [63]	Ghana	IS6110 and rpoB PCR	Genotypic clusters showed spatial aggregation
Zelner J, et al. 2016 [60]	Peru	24-MIRU-VNTR	Genotypic clusters showed spatial aggregation

*PGRS* polymorphic GC-rich repetitive sequence

Combinations of multiple methods were typically used for genotyping, with the commonest being IS6110 restriction fragment length polymorphism (IS6110-RFLP) and spoligotyping (*n* = 9), followed by mycobacterial interspersed repetitive unit variable number tandem repeat (MIRU-VNTR) and spoligotyping (*n* = 5), although use of a single method was reported in six studies (Table 4). No identified studies reported use of whole genome sequencing.

#### Temporal distribution of genotypically clustered cases

The temporal pattern of genotypic clustering could provide insights to distinguish between transmission and reactivation. In some studies, the temporal distribution of genotypically clustered cases indicated periods of 1 to more than 8 years between the genotypically clustered cases [1, 72], implying reactivation TB could also show genotypic similarity.

#### Use of spatial methods to inform public health interventions

In addition to their use in characterising the spatial distribution and determinants of TB, spatial methods have been used to inform TB-related public health interventions. In these studies, spatial analysis methods have proved to be attractive in guiding public health interventions, although their application to TB care beyond research is not well documented. For instance, spatial analysis techniques have been used to identify locations with a high density of TB cases (termed hotspots, although this definition was not based on spatial statistical tests). Community screening was then conducted in these areas, and its yield was compared to that from routine service provision. This GIS-guided screening was found to considerably improve the detection of individuals with latent TB infection and other infectious diseases [73]. Similarly, a study from South Africa highlighted the potential for using GIS to promote community-based DOTS by locating and geographically linking TB patients to their nearest supervision sites, although programmatic implementation of this approach was not reported [74].

The potential for spatial methods to be used for the early detection of TB outbreaks has also been described, although the findings widely varied based on the background population [1, 28]. Spatial cluster analysis using data at higher geographic resolutions improves the method’s performance in cluster detection [25].

## Discussion

While a range of methodologies has been employed in divergent contexts, we found that essentially all geospatial studies of TB have demonstrated significant heterogeneity in spatial distribution. Spatial analysis was applied to improve understanding of a range of TB-related issues, including the distribution and determinants of TB, the mechanisms driving the local TB epidemiology, the effect of interventions and the barriers to TB service uptake. Recently, geospatial methods have been combined with genotypic clustering techniques to understand the drivers of local TB epidemiology, although most such studies remain limited to low-endemic settings.

In almost all reviewed studies, retrospective program data (notifications) were used. Notification data, especially from resource-scarce settings, suffer from the often large proportion of undetected cases and are heavily dependent on the availability of diagnostic facilities [12]. None of the spatial studies of TB that used notification data accounted for undetected cases, such that the patterns in the spatial distribution and clustering could be heavily influenced by case detection performance [11]. Hence, distinguishing the true incidence pattern from the detection pattern has rarely been undertaken, despite its importance in interpretation.

The problems of undetected cases could be compounded in the spatial analysis of drug-resistant forms of TB, especially in resource-scarce settings where testing for drug-resistant TB is often additionally conditional on the individual’s risk factors for drug resistance [75]. However, recently, there have been some attempts to account for under-detection in the spatial analysis of TB. A Bayesian geospatial modelling approach presented a framework to estimate TB incidence and case detection rate for any spatial unit and identified previously unreported spatial areas of high burden [11]. Another approach is to estimate incidence using methods such as capture-recapture [76, 77] and mathematical modelling [78]. If case detection rate is truly known for a defined region, incidence can be calculated as notifications divided by case detection rate, although this is rarely if ever the case. Spatial analysis using prevalence data could also be considered in areas where such data are available.

In relation to the data problems outlined above, spatial analysis of TB could benefit from the use of model-based geostatistics, which is commonly used in other infectious diseases [79], although there are few studies that consider *Mtb* [80]. In particular, measurement of TB prevalence is impractical to perform at multiple locations due to logistic reasons. Therefore, model-based geostatistics can be used to predict disease prevalence in areas that have not been sampled from prevalence values at nearby locations at low or no cost, producing smooth continuous surface estimates.

Mapping of notification rates was the most commonly used data visualisation technique, in which TB cases were categorised at a particular administrative spatial level. This approach has the advantage of easy interpretability, although it can introduce bias because the size of the regions and the locations of their boundaries typically reflect administrative requirements, which may not reflect the spatial distribution of epidemiological factors [19, 22]. In addition, patterns observed across regions may depend on the spatial scale chosen, an effect known as the modifiable areal unit problem (MAUP) [17]. Because the choice of spatial scale mainly depends on the limitations of available data [81], only one study was able to provide a systematic evaluation of the effect of scale on spatial patterns, demonstrating improved performance of Kulldorff’s spatial scan statistic method at a high geographic resolution [25]. Different spatial resolutions could lead to markedly different results for the same dataset regardless of the true extent of correlation, due to averaging (aggregation effect) or other spatial processes operating at different scales [15, 17, 18]. Assessing the presence of this effect should be a priority for future studies using aggregated data in spatial TB studies.

Bayesian smoothing techniques can mitigate the problems of stochastically unstable rates from areas with small population [81], although such techniques were not widely used in the included studies and so false spatial clustering remains an important consideration. The less frequent use of rate smoothing techniques in the spatial analysis of TB could have various explanations, including lack of software packages that are easily accessible to the wider user (although GeoDa spatial software currently provides an accessible platform to people with limited statistical or mathematical backgrounds [82]). It may also be that most spatial analyses of TB are based on data aggregated over larger geographic areas from several years, such that the problem of statistical stochasticity may not be a major problem, although this was not explicitly discussed in the included studies.

In all studies that applied spatial cluster identification tools, TB cases were clustered irrespective of whether the setting was low or high endemic. However, in studies that incorporated more than one cluster identification method, areas identified as hotspots were not identical, with the extent of agreement between the alternative methods highly variable. This could be partly attributable to different methods testing separate hypotheses, such that these results may correctly support one hypothesis while refuting another. However, there is no consensus on how to interpret these findings appropriately and consistently [82, 83], and method selection did not typically appear to be based on such considerations [84, 85]. Thus, caution is required when considering interventions assessing clusters with one method only, as is frequently undertaken in TB spatial analysis [22].

Use of multiple cluster detection methods and requiring their overlap to represent a truly high-risk area is increasingly recommended [82, 84, 86]. However, this approach could also increase the risk of false-positive spatial clustering when different methods are used serially until significant clusters are observed [85]. Sensitivity analysis of spatial clustering [87, 88] and cluster validation using geostatistical simulations [23, 89, 90] can help identify robust clusters. While methods that adjust for confounding are generally preferred [91], further investigative strategies including data collection and cluster surveillance are required to validate an observed spatial cluster before introducing interventions [84, 85]. Although the focus of this study is TB, several methodological considerations outlined here would remain true for many infectious diseases.

In several studies, presence of spatial clustering or spatial autocorrelation in TB distribution was considered to reflect ongoing TB transmission, while its absence was taken to indicate reactivation [58]. Recently, molecular techniques have been combined with geospatial methods to understand the drivers of local TB epidemiology, although findings from these studies vary by country and the subset of the population studied. While spatial clustering of genotypically related cases was reported in several studies and likely reflected intense local TB transmission [61, 65], spatial clusters were dominated by genotypically unique strains in some studies, implying that reactivation was the dominant process [47, 72]. Hence, the combination of genotypic and geospatial techniques can improve understanding of the relative contribution of reactivation and transmission and other local contributors to burden.

Notwithstanding the general principles outlined above, not all spatial clusters of genotypically related cases will necessarily result from recent transmission, as simultaneous reactivation of remotely acquired infection and limited genetic variation in the pathogen population can also lead to genotypic similarity of spatially clustered cases [2, 92]. In some studies, the time between the first and last diagnosis of the cases in the genetic cluster ranged from 1 to more than 8 years [1, 72], suggesting that genotypic clustering could occur from spatially clustered reactivation. Similarly, limited spatial aggregation of genotypically clustered cases [72, 93, 94] and lack of epidemiological links between genotypically clustered cases in some studies may reflect migration of the human population over the extended time scale over which TB clusters occur [95], although casual transmission creating spatially diffuse clusters is an alternative explanation.

The extent of genotypic similarity between cases also depends on the discriminatory power of the genotyping method and the diversity of the pathogen population. Compared to whole genome sequencing, standard molecular genotyping (spoligotyping, MIRU-VNTR and IS6110) methods generally overestimate TB transmission with a false-positive clustering rate of 25 to 75% based on strain prevalence in the background population [92, 96]. The accuracy of these tests in distinguishing ongoing transmission from genetically closely related strains is very low among immigrants from high TB incidence settings with limited pathogen diversity [92, 97]. Thus, care should be taken when interpreting the genotypic similarity of cases among immigrant groups, as independent importation of closely related strains is possible. The frequent finding of more extensive genotypic than spatial clusters [71, 94] may reflect overestimation by the genotypic methods [98]. On the other hand, TB transmission might not result in apparent spatial clustering due to reasons that include population movement, poor surveillance and unmeasured confounding.

Regression models used for spatial analysis of TB were either conventional regression models or models that incorporated spatial effects. Although the former was more commonly employed, the majority of models incorporating spatial effects confirmed that accounting for spatial correlation improved model fit [11, 33, 44, 58, 99–101]. Conventional regression models assume spatial independence of model residuals and so ignore the potential presence of spatial autocorrelation, such that non-spatial models may lead to false conclusions regarding covariate effects.

The use of the conventional regression models described above may be appropriate for spatial analysis and spatial prediction, in the case that spatial dependence in residuals has been ruled out. Under this approach, the standard procedure is to start with classical ordinary least squares (OLS) regression models and then look for spatial dependence in the residuals, which implies the need for a spatially explicit regression model [82]. Several of the models reviewed here did not appear to adopt this approach, and so, caution is required when interpreting the findings from such analyses.

Most regression models treat the association between TB rates and ecological factors as global and are unable to capture local variation in the estimates of the association. However, geographically weighted regression (GWR) estimates coefficients for all spatial units included [22] and has often found the effect of risk factors on TB incidence to be spatially variable [16, 102–104], implying that global models may be inadequate to consider locally appropriate interventions. Few studies were able to perform explicit Bayesian spatial modelling incorporating information from nearby locations, thereby producing stable and robust estimates for areas with small populations and robust estimates of the effects of covariates [91].

While our review focused on methodological issues, several consistent observations were noted. Most importantly, all studies included in this review demonstrated that TB displayed a heterogeneous spatial pattern across various geographic resolutions. This reflects the underlying tendency for spatial dependence that can be caused by person-to-person transmission, socio-economic aggregation [49] and environmental effects [58, 93]. However, in nearly all included studies, spatial analyses of TB were based on the individual’s residence, although considerable TB infection is acquired from workplaces and other social gathering sites [8, 54]. Such studies could wrongly attribute TB acquired from such sites to residential exposure, leading to resource misallocation.

Several models have shown significant associations between TB rates and demographic, socioeconomic and risk-factor variables, although it is difficult to rule out publication bias favouring studies with positive findings. However, associations observed between TB rates and different factors such as population density, unemployment and poverty at the population level varied across studies. These were recognised as important individual-level risk factors, highlighting the potential for ecological fallacy.

We did not perform individual study level analysis of bias in this review. Analyses in the reviewed studies involved counts and proportions across different spatial distributions, rather than comparisons across different treatment/exposure groups. Standard tools of bias analysis predominantly focus on different treatment groups within cohorts (absent from our included studies) and hence are not applicable to this review. We have however discussed many potential sources of bias in the studies included in our review.

Most of the reviewed studies were from high-income settings, which may either reflect publication bias or a focus of research efforts on such settings. In high-incidence settings, the more limited use of spatial analysis methods could reflect a lack of access to resources (e.g. georeferenced data and spatial software packages) or insufficient expertise in these settings. However, it is these high-transmission settings which stand to gain the most from an improved understanding of TB spatial patterns and also these settings in which geospatial clustering may be most important epidemiologically.

## Conclusions

A range of spatial analysis methodologies have been employed in divergent contexts, with virtually all studies demonstrating significant heterogeneity in spatial TB distribution regardless of geographic resolution. Various spatial cluster detection methods are available, although there is no consensus on how to interpret the considerable inconsistencies in the outputs of these methods applied to the same dataset. Further studies are needed to determine the optimal method for each context and research question and should also account for unreported cases when using notifications as input data where possible. Combining genotypic and geospatial techniques with epidemiologically linkage of cases has the potential to improve understanding of TB transmission.

### Additional file

Additional file 1: **Figure S1.** Trends in the spatial analysis of TB (note—the study included publications up to February 15, 2017). (DOCX 17 kb)

## Appendix

### Search strings

#### Search terms used in Embase, Medline, PsycInfo, Scopus and Web of Science

The exp refers to explode which means include all subheadings underneath spatial analysis. When exploded, it contains geographic mapping, spatial regression and spatiotemporal analysis.

Brackets () denote subject headings (MeSH in Medline and Emtree in Embase) terms highlighted by the database.

#### Medline and PsycInfo

1. (exp spatial analysis) OR (Geographic information systems) OR (Space-time clustering) OR geographic* analys*.mp OR spati*regres*.mp OR spat*temp*.mp OR spat* analys*.mp OR spat* temp* analys*.mp OR spat* temp* pattern*.mp OR geography* distribut*.mp OR spat* temp* distribut*.mp OR heterogen* distribut.mp OR spacetime cluster*mp OR space-time cluster*mp OR hotspot.mp Or hot spots. mp OR GIS OR spati*
2. (tuberculosis) OR (tuberculosis, multidrug resistant) OR TB.mp
3. 1 AND 2

#### Embase

1. (spatial analysis) OR (geographic mapping) OR (spatial regression) OR (Spatiotemporal analysis OR (spatial autocorrelation analysis) OR (geography) OR (geographic distribution) OR (geographically weighted regression) OR (geographic information systems) OR (cluster analysis) OR geographic* analys*.mp OR spati*regres*.mp OR spat*temp*.mp OR spat* analys*.mp OR spat* temp* analys*.mp OR spat* temp* pattern*.mp OR geography* distribut*.mp OR spat* temp* distribut*.mp OR heterogen* distribut.mp OR spacetime cluster*mp OR space-time cluster*mp OR hotspot.mp Or hot spots. mp OR GIS OR spati*
2. (tuberculosis) OR (multidrug resistant tuberculosis) OR TB.mp
3. 1 AND 2

#### Scopus

(“Spatial analysis” OR

“Spatio-temporal analysis” OR

“Geographic Information System” OR

“Geographic Mapping” OR

“geographic distribution” OR

“spatial regression” OR

“spatial autocorrelation analysis” OR

“Spatiotemporal analysis” OR

hotspot OR

“hot spot” AND tuberculosis/TB

#### Web of science

[(Spatial analysis) OR

(Spatio-temporal analysis) OR

(Geographic Information System) OR

(Geographic Mapping) OR

(geographic distribution) OR

(spatial regression) OR

(spatial autocorrelation analysis) OR

(Spatiotemporal analysis) OR

(hotspot) OR

(hot spot)] AND (Tuberculosis)

## Abbreviations

CAR models: Conditional autoregressive models
GIS: Geographic information system
GWR: Geographically weighted regression
HIV: Human immunodeficiency virus
LISA: Local indicators of spatial association
NNI: Nearest neighbourhood index
PCA: Principal component analysis
TB: Tuberculosis
