# Supplementary material for: Methods used in the spatial analysis of tuberculosis epidemiology: a systematic review
Source: BMC Med. 2018 Oct 18;16:193. doi: 10.1186/s12916-018-1178-4 (PMC6193308; doi:10.1186/s12916-018-1178-4)
Supplement: Supplementary file 1 — Figure S1. Trends in the spatial analysis of TB (note—the study included publications up to February 15, 2017). (DOCX 17 kb) [file 12916_2018_1178_MOESM1_ESM.docx]

Figure S1: Trends in the spatial analysis of TB (Note- the study included publications up to February 15, 2017)
